# An Important Need to Monitor from an Early Age the Neurotoxins in the Blood or by an Equivalent Biomarker

**DOI:** 10.3390/ijerph16183425

**Published:** 2019-09-16

**Authors:** Keith Schofield

**Affiliations:** Materials Research Laboratory, University of California Santa Barbara, Santa Barbara, CA 93106-5121, USA; KSHome@ucsb.edu; Tel.: +1-805-966-6589 (ext. 451-7996)

**Keywords:** neurotoxins, genetic susceptibilities, baseline values, inoculation pretesting, vaccine management, minimum risk levels, personal medical responsibility, national body-biomonitoring

## Abstract

An overwhelming amount of evidence now suggests that some people are becoming overloaded with neurotoxins. This is mainly from changes in their living environment and style, coupled with the fact that all people are different and display a broad distribution of genetic susceptibilities. It is important for individuals to know where they lie concerning their ability to either reject or retain toxins. Everyone is contaminated with a certain baseline of toxins that are alien to the body, namely aluminum, arsenic, lead, and mercury. Major societal changes have modified their intake, such as vaccines in enhanced inoculation procedures and the addition of sushi into diets, coupled with the ever-present lead, arsenic, and traces of manganese. It is now apparent that no single toxin is responsible for the current neurological epidemics, but rather a collaborative interaction with possible synergistic components. Selenium, although also a neurotoxin if in an excessive amount, is always present and is generally more present than other toxins. It performs as the body’s natural chelator. However, it is possible that the formation rates of active selenium proteins may become overburdened by other toxins. Every person is different and it now appears imperative that the medical profession establish an individual’s neurotoxicity baseline. Moreover, young women should certainly establish their baselines long before pregnancy in order to identify possible risk factors.

## 1. Introduction

Neurotoxins are becoming a very major component in our lives, yet most people as well as the medical profession mainly disregard them. As a result, it is imperative to examine the current situation and possibly take more responsibility for our own well-being, which now seems to be controlled by innumerable factors, including our living conditions, lifestyles, diets, drinking water, and genetic susceptibilities. These factors are generally not well documented by our personal doctors, who unfortunately still have to depend on symptoms to a large degree. Other than the major field of vaccines, the concept of preventive medicine is only now coming more to the forefront with the current possibility of additional testing procedures. A major factor that goes largely unquestioned, and not seriously considered, is that humans are all different. The consequences of individual variation complicates modeling and is displayed as distributions in any general statistical analyses of medical data [1]. The object of this assessment is to stress that it is really important to know where an individual lies on such toxicity distributions and whether they are in fact living with a significant medical risk.

## 2. The Major Neurotoxins

From conception until death, the neurons of the brain survive by having significant protections. Nevertheless, the brain is at constant risk, especially in this day and age where the environment is constantly changing and introducing new risks. Such dangers can result from the myriad of natural or man-made chemicals that may pass through the blood–brain barrier, causing genetic damage [2,3]. About 70 years ago, Thalidomide was one of the first organic pharmaceutical medications that caused major birth defects, mainly in Europe, and was soon identified and banned from human use. Other organics posing risks for pregnancies were also identified, listed by the medical profession, and are now similarly avoided [4]. Fortunately, the human body appears to process most of this myriad of organic chemicals, many of which are hazardous and may induce symptoms, but are often related to cancer rather than being neurotoxic. Recently, an interesting review of neurotoxicant chemicals was published based on innumerable rodent tests due to the ever-present difficulty of using human volunteers [5]. This has various disturbing neurotoxicant entries such as caffeine, cocaine, ethanol, fluoride, heroin, nicotine, and ozone, which are encountered regularly but still have lifetime consequences that are unknown. However, what is now apparent, and being confirmed by experiments, is that numerous inorganic elements and their chemicals are found on the list and are known to be common in the human diet, in the living environment, or are in medical use, clearly neurotoxic, and are not as easily evaded or discounted [6,7]. Fortunately, these are only a few in number, but they can pass through the blood brain–barrier and also the placenta of pregnant women. These are the major concerns of this article and current research, namely: Aluminum Al, arsenic As, lead Pb, mercury Hg, manganese Mn, and selenium Se. The first four are commonly found in the human body, yet serve no purpose whatsoever and constitute a burden that the human body has to constantly address and manage. Research on these has been extremely extensive during the last two decades and although their relative concentrations in the environment may vary geographically, global exposure is ubiquitous, in particular, for the molecular compounds of lead, mercury, and arsenic. Aluminum has gained importance in recent years since becoming the major adjuvant in vaccines, largely replacing mercury in US domestic use and being inoculated directly into the body. Manganese is required by the body and is now becoming examined in much greater depth [8]. As it is necessary, the body in most cases should normally regulate it. Deficiencies and excesses tend to be rare, but are suspected in controlling infant birth weights [9] or may lead to neurological illnesses [10,11,12,13]. Selenium has emerged as the safeguard, being the body’s main chelator and brain cleanser, particularly for the four intruders [14,15,16]. A consensus has now emerged that these elements have the potential to be major components in the spectrum of neurological illnesses that are now at epidemic levels. After much study, it has also become apparent that single causes are unlikely and evidence is mounting that the synergistic effects between two or more contributors, coupled with genetic susceptibilities [17,18,19,20], are far more likely.

## 3. Global Surveys of Body Chemistry

The advent of advances in analytical ability opened the medical door to understanding the body’s chemistry to a fuller extent. Surveys of populations worldwide have provided, for the first time, knowledge of the chemical composition and particularly concentrations of trace substances in the human body, their distributions, and histories. To aid understanding, much time has been spent establishing the preferred biomarkers that can become meaningful measures of the body’s chemistry. The surprises that have arisen confirm the differences and also similarities globally between people. Another surprise has been just how many elements of the periodic table and organic compounds manage to be ingested by human diets [21,22,23]. The human body needs about 15 elements, Na, Fe, Ca, Mg, and K being major elements, ranging on average, in the blood, between about 30 gm/L in order down to 40 mg/L, followed by Zn, Rb, Cu, and Se at about 8 down to 0.3 mg/L and Mn, Mo, Li, Cs, Co, and Cr(III) at about 12 down to 0.9 µg/L [24]. Other than traces of Cs, molybdenum Mo at atomic number 42 in the periodic table is the heaviest metal, and all the others are from the upper part of the periodic table. Even 30 years ago, in the infancy of such testing, an Italian study measured 46 elements in urine, blood, and serum from 350 healthy people. Currently, 17 more could be easily added to their list [25]. A more recent survey in France of 106 healthy volunteers monitored 27 metal elements in blood samples [26]. Elements that still have questionable roles in the body are As, B, Ni, Si, and V, but are at very low µg/L concentrations. All others are non-essentials but can be consumed in certain diets and are on low µg/L scales in blood concentrations. Studies still continue investigating all these trace elements in the body and their possible interactions, but are only of interest in comparisons between cultures [27,28]. Other recent research has measured the hundreds of organics present at low levels in blood [29]. All have to be cleansed from, or maintained by, the body. As a result, to claim any single element as being responsible for some particular consequence in the human body is an impossible suggestion for the medical profession because there are so many variables. In other words, attempts to correlate the cause of any illness using statistical analyses of biomarker data relating to one, or even two aspects, is not scientifically plausible and always has to remain speculative. Especially for epidemics, which are becoming evident in a broad spectrum of neurological illnesses, a different approach is obviously necessary. One new approach was previously outlined in a discussion of potential causes of autism [30]. It is common sense that, if an epidemic becomes apparent, it indicates that some change has occurred that triggers the growth. Consequently, a more fruitful examination would be to explore what actually has changed. For a human case, this entails examining changes in life-style, living conditions, dietary changes, medical services, and global geographic occurrences. Global epidemics have a demanding list of necessary criteria that immediately eliminates many suggestions due to such strict requirements.

## 4. Distributions and Toxic Limits

**Figure 1:**
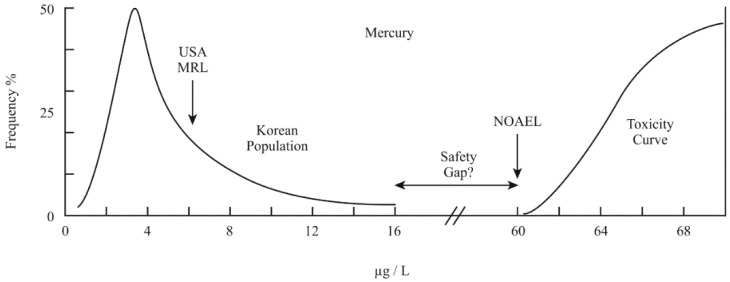
Distribution of blood mercury levels in a Korean population [31,32] compared to the USA MRL level (minimum risk level) and the NOAEL (no observable adverse effect level) toxicity curve onset, µg/L.

Biomonitoring is useful to indicate the content of a certain chemical concentration in the body. If it is a toxin or neurotoxin, then it can be compared and appraised against the expected human concentration range and any suggested safe level (MRL minimum risk level), which is often based on its known or estimated NOAEL concentration (no observable adverse effect level). This appears straightforward, but it enters into the field of toxicology where numerous assumptions are made and are now beginning to be questioned. Figure 1 illustrates this basic concept for the case of mercury neurotoxicity. From many surveys of populations, this one has been chosen randomly to illustrate these assumptions and difficulties. It is based on two recent similar Korean measures of mercury in whole blood from 260 and 4000 healthy individuals [31,32].

With any survey of a neurotoxin distribution of people, a significant range of values is always obtained. A number of these have been published before in order to display the expected distributions for these six neurotoxins [33]. They all are heavily weighted to lower values, but beyond 50% of the samples, the distribution becomes characterized by a thinning range of values that can gradually stretch to a much larger extent. A small fraction of significant values beyond 98% of the population are invariably obtained, generally called outliers. These are for individuals where the value possibly results from accumulation and retention. For individual elements, the basic shape is generally similar, but can vary in peak location and spread, depending on geographic and genetic factors. However, as noted in Figure 1, it is obvious that higher values are in the direction of toxicity and greater risk. One aspect of the surveys is that they aim to establish average values. They give no indication of how an individual’s value may vary with time. It undoubtedly has a range and might elevate significantly after a visit to the doctor or after a banquet dinner with fish. Such spiked values are never discussed, but may be in possible times of excesses the slight accumulations of damage that may occur through life. The assumption of no damage from low levels is now being extensively questioned [34,35,36]. Fortunately, it was noted that when all these surveys were obtained, the values were not specifically encroaching into the estimated toxicity curves and helped to suggest the minimum risk levels 100-fold below the known NOAEL onsets in some cases. Mercury is at some risk with the suggested US MRL (minimum risk level) value being only 10-fold below the toxicity onset. In this case, the NOAEL is a reasonably fixed value based on human data, and to reduce the risk requires that the population modify its intake behavior. The general present policy is based on the concept that, if no response or symptom is noted, all is well. In other words, does the apparent safety gap in Figure 1 have a real meaning? This assumes that the body can manage certain, low levels of toxicants with no harm. However, once in the body, the toxicant is active so whether traces of damage can arise remains unknown. Additionally, for most neurotoxicants, it is especially difficult to establish accurate human NOAEL values as they often have to be extrapolated from rodent studies. Uncertainties will remain in this conversion to the suggested MRL levels. In the case of mercury, the US approximate value was obtained directly from extensive studies of, largely, fish-eating human populations. The accepted view is that the body has built in protections against minor risks that cause no observable damage. However, the currently suggested safe intake of lead is now regarded as zero, which is generally not attainable and a higher limit becomes the goal, even though long-term ramifications are apparent and expected [37,38]. In fact, accumulations over a lifetime of even small amounts cannot be discounted. Epidemics now have changed the course of medical research in recent years and numerous previous concepts are being modified. One recent survey of about 1000 participants in New Zealand for blood lead levels was repeated after a period of 38 years and found continued adverse changes in IQ (intelligence quota) scores after such a period [39].

## 5. Current Insightful Research

The average typical concentrations that can be seen in human blood are interesting. The four alien elements, not needed by the body, are in low concentrations of about 5–15 µg/L (Al, As, and Hg) with Pb hopefully at about 25 µg/L, but this is invariably controlled by environmental circumstances. Manganese is needed in small quantities by the body, about 15 µg/L, yet selenium is invariably seen to be much larger than these at about 100–300 µg/L. Research has now confirmed that there is meaning to this. In recent decades, selenium has come to the forefront and has been noted as a very major natural body and brain chelator. It forms innumerable proteins such as selenocysteine and selenomethionine, which can attach, sequester, and transport from the brain the four alien neurotoxins that are of particularly interest in this study. These are known neurotoxins and can damage the brain. This role for selenium, concerning neurodegenerative diseases, has been suggested for a number of years and is now becoming fully accepted [40,41,42]. It has been acknowledged as being especially important in pregnancies [43,44,45,46]. What remains uncertain is selenium’s efficiency and chemical rates of interaction with the alien toxins. What has been measured, generally by isotopic labeling, are the half-live’s of such elements in the body and brain, generally utilizing rodents. It is obvious that the longer these elements are in the body, the greater is the probability of not only increased transfer across the blood brain barrier, but also a specific period in the brain causing possible damage. For Al, an extensive review [47] summarized this earlier work. Inoculations showed 100% body absorption in comparison to only less than 1% for ingested forms. As with many cases, elimination varies depending on where it concentrates in the body. In this case, 85%–90% was found to be eliminated in <1 day, 4% remaining after three-years, and 2% after nine-years. Half-lives connected to these measures indicated values of about 1.4, 40, 1727 days, and about 50 years. There appeared to be a slight increase with age of Al in the brain and its half-life was indicted as >100 days in one study and estimated as ≈20 years in another. Irrespective of their accuracy, such measurements confirm the simple aspect that most neurotoxins, once in the brain, are slow to egress. Whether they are sequestered or remain active during their lifetime remains uncertain. A recent brain tissue study suggested that Al has a putative role in autism spectrum disorder, but this remains speculative [48]. Arsenic is cleared quickly from the blood with a half-live of 40–60 h or less [49]. A blood MRL of about <15 µg/L is suggested by several testing centers. It is not significant in brain autopsies and little is known of its concentrations in the brain, other than that transfer rates appear small [50]. This may be one neurotoxin that has a limited time in the body in which to seriously access the brain. However, in areas of high As in drinking water, such as Bangladesh, high rates of cancer are noted in long-term exposure [51,52]. It appears to be more of a toxin for the body rather than creating neural problems. Lead is known for its toxicity, even at low levels, and has been extensively studied [53,54,55]. No MRL is suggested as a safe level is regarded as zero [38]. Blood half-life in the body is quoted as 20–30 days, however this is more difficult to assess because non-negligible levels are always present. Desirable blood level concentrations now are tentatively suggested as <50 µg/L, but this is still exceeded in many parts of the world and is a wishful target. Low-level lead exposure does highlight genetic susceptibility, especially in children [56]. It is reported in brain autopsies. Levels are always present in the body, so there is adequate time for transfer into the brain. The half-life remains uncertain for Pb, but neural effects are commonly observed in contaminated areas. A recent study with rats had the intriguing conclusion that lead toxicity induced in youth, declined with age, but reemerged in older-age rodents [57]. Whether this extrapolates into humans might imply that early damage is life-long and is to be avoided. While lead use is being phased out, lead is still in paint at levels of 1% in many parts of the world [58]. Mercury is possibly the most studied of the elemental neurotoxins. Its greatest risk is posed by its organic forms, the methyl-mercury found in nature, in fish, and now rice, and Thimerosal, an ethyl form (sodium ethyl mercury thio-salicylate), remains to be the adjuvant in some vaccines. Studies of high fish eating diets in the US suggested an MRL for mercury blood levels of 5.8 µg/L. This is about 10-fold below its NOAEL of 60 µg/L, as noted in Figure 1. It remains marginally compatible with the US population’s distribution, but mercury toxicity effects are now quite common. Some countries are suggesting a lower level such as 2 or even 0.8 µg/L, which is an admirable goal if it is consistent with the population’s diet and life-style. Carbon radiolabeled studies on animals indicated, for ethyl-mercury, a full body half-life of seven days and a brain half-life of 24 days, these values varied to 19 and 60 days, respectively, for methyl-mercury. In a very extensive study, they also indicated that, although the ethyl form concentrations were 3-fold lower, it de-alkylated in the brain to the mercury ion, over the methyl form by a larger fraction of 34% vs. 7%, making its egress more difficult. This behavior was confirmed in both rats and monkeys [59,60,61]. Obviously, with such half-lives, the brain is certainly at a high risk of damage, depending heavily on the efficiency of the Se proteins to nullify their presence. Low doses of methyl-Hg, equivalent to those found in the human diet, when fed to rats, were shown to produce noticeable neuro-degenerative effects [62]. No MRL or NOAEL values have been suggested for manganese [63]. Measurements with rats appeared to show a continuing absorption by the brain of labeled Mn and slow egress [64,65]. As it is essential to the body, it may be automatically controlled. However, there is an implication that birth weight might be influenced by its excess [9]. Several studies with labeled Mn indicate that it can accumulate in the bone and have either a somewhat fast elimination measured in days or a slower process measured in years [66,67,68].

There is now a realization that some neurotoxins can stay in the body for a significant time and can cause adverse effects, even at low levels that were previously thought to be safe [69]. Recent research confirms that doses below the suggested safe toxicity levels can indicate effects with time; a new so called “silent toxicity” [70,71]. The concept of “no symptom, no concern” may need reassessing, and neurotoxins may be detrimental without indicating diagnostic diseases [72]. In addition, the question of cumulative synergistic effects is only now being addressed, but is apparent [73]. One concern in areas of heavy industry is the evident epidemic of neural-tube defects, as well as high rates of miscarriages and preterm births. It raises the question of whether Se body-levels are too low or whether this reflects an overburdening of the selenium enzyme-formation rates, that are becoming rate-limited, as is currently being suggested by many [46,74,75,76,77,78,79,80,81]. Experiments with rats record long-lasting impairment even after prenatal exposure to CH_3_Hg [82].

Pregnancies have been a major concern in regards to toxicity for decades, especially when observing toxicity levels far in excess of the body weight of a fetus. It has been suggested that there are possibly no safe levels with neurotoxins for the fetus due to its small size [83]. This has been more strongly presented in actual analyses of the neurotoxin levels being experienced by a fetus and is a long standing unanswered quandary [84,85]. As toxicity is expressed by a unit concerning body weight, the low weight of a fetus or baby reduces the safe blood level value drastically because a fetus at eight weeks has a mass of 1 g. This has raised the ignored question of how any fetus is born alive. In this regard, two recent publications may be of great importance. It is well documented that the placenta is a poor filter for controlling metallic elements [86]. As a result, no explanation has ever been presented as to how a small fetus manages to survive such a barrage of neurotoxins, other than that it possibly has a strong blood–brain barrier and other still unknown defenses. This is especially noteworthy in regards to the otherwise self-protecting mechanisms throughout the body and the intricacies of the DNA survival modes. Currently, two studies have emerged that measured the actual selenium and mercury levels in the placenta and umbilical cord at birth in 91 Polish cases and 54 Japanese [87,88]. In one case, they found that, on a molar ratio, the Se was in excess of mercury by ratios from 6–650 and 21-fold in the other. Such an excess of selenium over the neurotoxins may be the unknown protective mechanism. Other reports of cord blood levels also show this excess of selenium [89,90]. In a detailed study, rats were fed CH_3_Hg, CH_3_Se, or a mixture of the two, together with controls [91]. Those on the mixture of Hg and Se were seen to be unaffected and showed no neural damage; they were apparently totally protected by the Se. It appears possible that the organic proteins of selenium are not only protective in nature, but may also facilitate the neuronal repair of any DNA damage [92]. Another unanswered question to consider is whether genetic susceptibilities moderate with age.

## 6. Recent Dietary and Medical Changes

Human medical epidemics, if not infectious or contagious, largely relate to the ingestion of food, water, or toxins. The neurotoxins are readily available from food, commercial products, and water quality. The examination of changes for these in the last 25 years shows very few that are global in nature. Trace metals have an anthropogenic nature and thus are open to regulation and mitigation [93]. In fact, legislation has been imposed on several neurotoxins during previous decades and their concentrations in the environment have decidedly decreased, particularly in the West. A major example has been for lead, largely removed from leaded gasoline, paint, plumbing fixtures, leaded crystal glass, and bullets, and now it is in significant decline. Arsenic can exist in natural drinking water, but is tightly monitored in developed Western countries, but remains in excess in many other regions of the world. An increase in aluminum is noteworthy. This has become the major adjuvant now in most domestic US vaccines and is common in some countries’ soils and dust. Mercury has decreased in most US domestic vaccines and has been removed from all commercial activities. Nevertheless, due to previous historical uses, levels in the environment remain high, especially when combined with the ongoing combustion of fossil fuels, which is not yet regulated globally. In addition, the rapid new growth of the sushi Japanese fish-style of foods has substantially increased the intake of organic methyl-mercury from fish consumption. In addition, Asian countries are now concerned with both As and CH_3_Hg contamination of rice [94,95,96,97,98]. All foods generally have some form of low concentration distribution of trace metals. The question is no longer whether one metal poses a medical problem, but what is the total collaborative sum ingested/injected. An additional complexity is of course their chemical forms (speciation) that may be toxic to varying degrees [99,100]. The organic forms of As in fish can normally have low, if any, toxicity because they are not metabolized by the body. However, the recent findings of arsenolipids in fish and even nursing milk has modified this view [101,102]. Additionally, the fact that fish can have a selenium content capable of countering its mercury or other neurotoxic content to varying extents now poses a severe problem for doctors and their dietary recommendations for pregnancy. Moreover, it has become a complex risk assessment even for the general population [103]. In fact, the concept of Se “deficiency risk” is becoming more important with respect to nutrition [104,105,106].

## 7. The Current Role Now Played by Vaccines

Programs of vaccinations are considered to be one of the major triumphs of modern medicine. That is certainly unquestioned. Nevertheless, it has to be accepted that, due to their nature and the fact that people represent a distribution of genetic susceptibilities, there has always been a low and accepted risk attached to any vaccine. The mechanisms of action for most adjuvants remains poorly understood and vaccines become accepted after extensive testing when adverse effects, and even death rates, become acceptably small. However, to indicate that they are scientifically proven to be safe is not true, and their nature, coupled with the complexities in the body, will never make the claim valid. They actually come based on the fact that “some will be sacrificed for generally unknown reasons for the greater good of the population”. Numerous excellent books have been written on the adverse effects of vaccines and the difficulties of their assessment [107]. Currently, most people are inoculated with little or no pre-knowledge, whether they are genetically susceptible or immune defective remains unquestioned. Before 1990, the number of inoculated children in the US was three to four-fold less than now. Since 2000, inoculation programs have grown extensively, now covering about 16 diseases in a programed regimen through to young adults. The current rate is twice that of other advanced countries and now undoubtedly requires management modifications. The program still contains a policy of inoculating pregnant women for influenza. This received a significant jolt when it delivered two mercury containing flu vaccines to pregnant women in the 2009/2010 season. The reported level of spontaneous abortion/stillbirths indicated a magnitude increase over the prior or later season and was one reason for removing Hg (Thimerosal) from vaccines as the adjuvant [108]. It clearly indicated that some mothers were close to the limit of acceptance. In fact, it is now reported that levels of As, Hg, and Pb are a concern for many pregnant women around the world [109,110]. All women are seen to have significant baseline levels of all toxic metals [28,111,112]. Neurotoxin levels are the most important during pregnancy and for the first few years with children. This is when fetal brains are still immature and growing. Neurotoxin levels for adults differ, partly through increased body mass, but also through more difficulty in damaging a mature fully formed brain. What is becoming clear is that vaccines truly may be safe if they are the only consideration. However, times have changed, and with monitoring, it is now apparent that other considerations have to be included. It has also become an example of “you can have too much of a good thing”. The fact that everyone is different, particularly in their medical genetic susceptibilities to illnesses, is largely ignored by the vaccine inoculation programs. It would appear now, based on all the biomonitoring surveys, that this can pose a risk to some people. Vaccine neurotoxicant adjuvants may become too much when added to people’s lifestyles. They should be entitled to know what their situation is before being forced into a mandatory vaccine program for both themselves and their children. Those known to already have high backgrounds of neurological elements may be automatically at risk of becoming overloaded. It would seem imperative now, if the medical profession is to restore general faith in vaccines in general, that those in the community that feel they are at risk (on high fish diets) should initially be free to be tested to establish their neurotoxin levels. This is especially appropriate for young women to establish their baseline values at an early age, certainly long before pregnancy. An analysis of the USA NHANES 2007–2012 National Surveys of 7408 individuals >6 years old, examining Pb, Cd, Hg, and As, found that half of them had at least three or more of these metals in their bodies [113]. Additionally, much data for pregnancy with maternal and fetal exposure is available, now indicating high-risk levels [114,115]. Additionally, the effects of potential long-term risks are not known for any neurotoxin and remain a suspect for contributing to age-related neurodegeneration such as Alzheimer’s disease [116].

## 8. Conclusions

The volume of medical research that has been published on the risks associated with the neurotoxins and their possible involvement in neurological illnesses is huge. It has arrived at a point where indications are now evident and future research has to be more specific, rather than repeating similar data. In this regard, measurements should involve the monitoring of a broader representation if any meaningful results are to be obtained. Solely measuring one species is pointless. The body is highly complex chemically and it is quite clear that its balance can be disturbed, often with resulting consequences, some of which may remain unknown and unnoticed. The important conclusion is accepting that all people, although apparently similar, are in fact all slightly different genetically and chemically. This is reflected in all medical testing when distributions are observed and statistics are used to establish average behavior. This is then used as a gauge and being average is “good”. This is also the situation with alien neurotoxins in the human body. Some people can discard them, while others not. Individuals have never before been particularly concerned over where they are on such distributions, but it now appears that this is becoming important. Additionally, although the body does have mechanisms for controlling neurotoxins, it remains unknown whether there is a price to pay for unnoticed, long-term low “safe” levels that go unnoticed. With personal involvement, monitoring and minimizing these background levels might lengthen and benefit one’s life significantly in medical aspects. Such concepts are already beginning to enter lives with cholesterol, sugar levels, and blood pressure monitoring, and now tends to suggest that neurotoxin level testing may become necessary to minimize neurological illnesses from autism across the spectrum to long-term old-age diseases. To facilitate this, a simple blood test is being encouraged for the general public, prescribed by their doctor that would simultaneously monitor the six neurotoxins in a small blood sample utilizing an ICP/MS (inductively coupled plasma-mass spectrometer) and compare this to average minimum risk levels [117]. This, for the first time, would establish an individual’s baseline levels and enable responsibility and management possibilities with greater confidence and lessen strain knowing one’s condition. It is already proven that it is possible and validated as a simple test utilizing an ICP-MS (Inductively Coupled Plasma-Mass Spectrometer) instrument in the US Centers for Disease Control and in China, but is not yet available for individuals or the general public [118,119]. The analysis of such a test would begin by assessing the probable sources for each element level and whether these could be readily mitigated. The goal would be to minimize each as much as possible. Beyond that, statistical programs would be invoked to assess the overall risk from such a mixture of levels. Such developments are currently being addressed by research examining multiple neurotoxin effects and also mathematical frameworks that can handle such complex mixtures [120,121].
